# Bile acids extend longevity beyond calorie restriction

**DOI:** 10.18632/aging.100175

**Published:** 2010-07-16

**Authors:** Antoine E. Roux, Pascal Chartrand

**Affiliations:** ^1^ Department of Biochemistry and Biophysics, University of California, San Francisco, San Francisco, CA 94158-2517, USA; ^2^ Department of Biochemistry, Université de Montréal, Montréal, Québec, H3C 3J7, Canada

The discovery of potential anti-aging treatment capable of extending both our healthy aging and perhaps our longevity resuscitated an old human dream. It also brought about inordinate interest in these studies. While calorie restriction (CR) is still the best known intervention to increase life span, anti-aging molecules could mimic this effect without reducing nutrient intake. Such compounds, like rapamycin and spermidine, believed to be CR mimetic, have been discovered using the yeast Saccharomyces cerevisiae. The interest in such molecules stems from their universal action on others species including mammals [1]. In this issue of Aging, a study by Goldberg and colleagues presents an original screen designed to isolate molecules that further lengthen the life span of yeast under calorie restriction rather than imitating this effect [2].

 In yeast, CR can be obtained by decreasing glucose concentration in the media. This intervention extends significantly chronological life span, i.e the time a yeast population remains viable in stationary phase [3]. By knocking-out peroxisomal proteins import (using a pex5Δ strain), the authors impaired beta-oxidation. As a consequence, the effects of CR on longevity are entirely abrogated. This genetic background was used to screen for chemical compounds that restore long life span under CR condition by targeting lipid metabolism. Among the chemical compounds identified, the authors focus on one group representing 6 bile acids compounds, the most efficient of them being lithocholic acid (LCA). Bile acids are mildly toxic oxidized derivatives of cholesterol that play important roles in lipid uptake by the intestine. Interestingly, none of the previously known small molecules that increase chronological life span in yeast (rapamycin, spermidine and methionine sulfoximine) were among the compounds that rescue the short longevity of pex5Δ under CR.

While the effect of bile acids on life span was identified in a pex5Δ strain, LCA also extends the life span of wild type yeast by over two fold in glucose restricted medium as compared to regular medium. This observation suggests that LCA acts on a substrate active during calorie restriction. Although the mechanism by which bile acids increase life span is still unclear, Goldberg and colleagues provide several clues that shed light on this phenomenon. Among them, LCA treatment improves triacylglycerol-dependent lipid storage, increases stress resistance, and improves mitochondrial fitness. Using genetics, the authors found that the target of LCA is most likely inhibited by Tor1 signaling and partially regulated by cAMP/PKA/Rim15, two pathways known to regulate aging rate in yeast [4,5]. However, as yeasts do not synthesize bile acids, the mechanism of action of LCA has yet to be elucidated.

Indeed, this study raises several interesting questions. Does LCA act as a pharmacological agent by binding a specific receptor in yeast or does it increase longevity by stimulating stress response due to its mild toxic effect (by hormesis)? Another question is whether bile acids or other related molecules can play an anti-aging role in metazoans. Current evidence from long-lived Little mice (Ghrhrlit/lit) and C. elegans proposes a role for bile acids in promoting longevity through a nuclear receptor mediated signaling [6,7]. If these xenobiotic molecules act through a universally conserved pathway from yeast to metazoans, it would make S. cerevisiae an unexpected model of choice to unravel this mechanism. Goldberg and colleagues open interesting and promising research to look for related compounds whose effectiveness would potentiate the longevity extension by calorie restricted diet. Studies in mammals will represent the real litmus test.

## References

[1] Harrison DE (2009). Nature.

[2] Golberg AA (2010). Aging.

[3] Fabrizio P, Longo VD (2003). Aging Cell.

[4] Kaeberlein M (2005). Science.

[5] Wei M (2008). PLoS Genet.

[6] Amador-Noguez D (2007). Aging Cell.

[7] Gerisch B (2007). Proceedings of the National Academy of Sciences.

